# A prenatal case of Simpson-Golabi-Behmel syndrome type 1 with a 0.26-Mb deletion fragment at Xq26.2 inherited from mother: Case report

**DOI:** 10.1097/MD.0000000000029222

**Published:** 2022-04-22

**Authors:** Jing Sha, Fangfang Tan, Ying Liu, Zaochun Xu, Xuezhen Wang, Jingfang Zhai

**Affiliations:** Xuzhou Central Hospital, Xuzhou Clinical Schools of Xuzhou Medical University and Nanjing Medical University, Xuzhou, Jiangsu, China.

**Keywords:** case report, copy number variation sequencing, glypican-3, prenatal diagnosis, Simpson-Golabi-Behmel syndrome type 1

## Abstract

**Rationale:**

The purpose of this report was to explore how to manage the fetus of Simpson-Golabi-Behmel syndrome type 1 (SGBS1) and to provide a definite diagnosis to guide the following genetic counseling for the pregnancy.

**Patient concerns:**

A 24-year-old women, gravida 1, para 0, was 172 cm tall with weight 65 kg. She was referred to our center for counseling due to second-trimester ultrasound screening anomalies at 22 + 5 weeks of gestation age. Meanwhile the ultrasound examination indicated the overgrowth of the fetus. She and her husband were healthy and nonconsanguineous without family history.

**Diagnoses:**

The karyotype and copy number variations sequencing (CNV-seq) combined with fetal ultrasound manifestation confirmed the diagnosis of SGBS1.

**Interventions:**

No treatment for the fetus.

**Outcomes:**

Pregnancy was terminated.

**Lessions:**

Once fetal overgrowth and other malformation are revealed in prenatal ultrasound, although without polyhydramnios and organomegaly, SGBS1 should be considered and further genetic testing such as CNV-seq and whole exon sequencing should be conducted to help clinicians provide a definite diagnosis to guide the following genetic counseling and the next pregnancy.

## 1 Introduction

Simpson-Golabi-Behmel syndrome type 1 (SGBS1, OMIM 312870) is an X-linked recessive disorder with the typical features of SGBS1 including pre/postnatal overgrowth, multi-system abnormalities such as cardiovascular defects, abnormal craniofacial features, genitourinary defects, and skeletal anomalies, as well as increased risks for embryonal tumors.^[1,2]^ It is the common one of the two subtypes associated with mutations of glypican-3 (GPC3) and glypican-4 (GPC4),^[3]^ which is usually diagnosed postnatally. Recently, sequencing technology has been widely applied to prenatal diagnosis and helped clinicians further define the genetic diagnosis associated with fetal structural abnormalities. Here, we report a fetus with overgrowth, congenital heart defects, and kidney defects. A 0.26-Mb deletion involving exon 3 to 6 of the GPC3 gene was identified in the fetus by Karyotyping and copy number variation sequencing (CNV-seq) through amniocentesis, meanwhile the genetic analysis of proband family members was conducted to confirm the mutation from the mother. Once overgrowth, abnormalities of multiple systems are observed in prenatal ultrasound, further genetic analysis can help to make definite diagnosis to guide the following genetic counseling.

## 2 Case report

**Figure 1:**
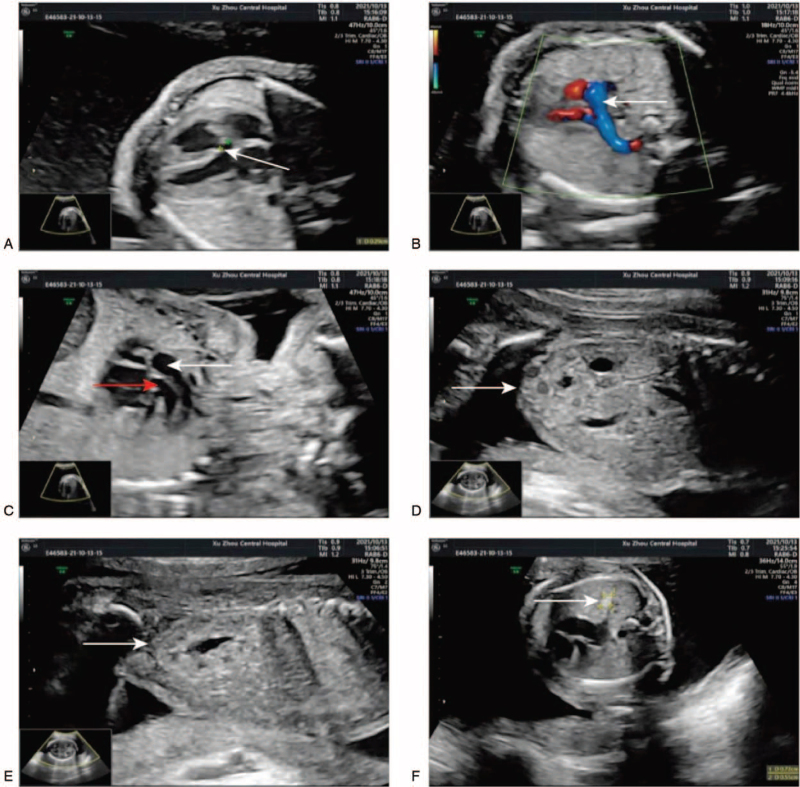
Prenatal ultrasound results: (A) ventricular septal defect, (B) aorta predominantly from the right ventricle, (C) the pulmonary artery (red arrow) originates in the right ventricle and runs parallel to the aorta (white arrow), (D) multiple cysts of the right kidney, (E) hyperechogenic kidneys, and (F) hyperechoic mass in the right lung.

**Figure 2:**
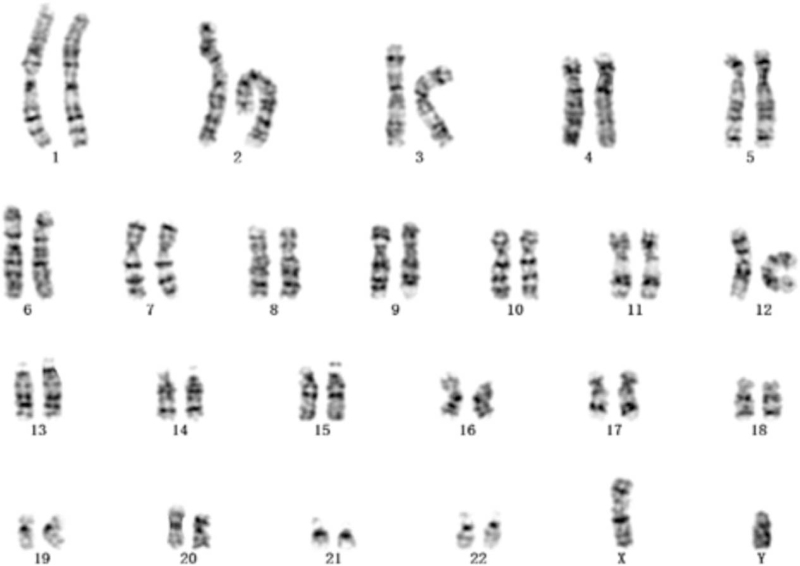
The fetal karyotype was 46,XY at the level of 300 to 400 bands.

**Figure 3:**
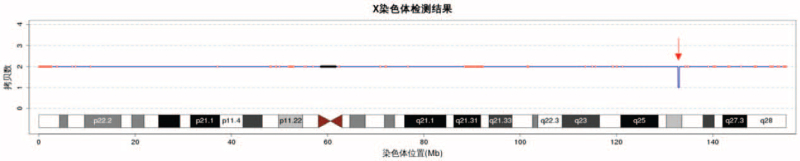
The result of CNV-seq analysis. A 0.26-Mb deletion at Xq26.2 (132800000–133060000). CNV-seq = copy number variation sequencing.

A 24-year-old healthy women, gravida 1, para 0, was 172 cm tall with weight 65 kg. She was referred to our center for counseling due to her second-trimester ultrasound screening anomalies at 22 + 5 weeks of gestation age. The ultrasound examination indicated the overgrowth of fetal: fetal estimated weight 743 g was in 99.9 percentile (+3.28 SD), biparietal diameter 62 mm in 98.9 percentile (+2.27 SD), head circumference 227 mm in 99.8 percentile (+2.89 SD), abdominal circumference 212 mm, in 100 percentile (+3.6 SD), the femur length 42 mm in 98.5 percentile (+2.16 SD). In addition to overgrowth, ventricular septal defect (VSD) (Fig. 1A), double outlet right ventricle (Fig. 1B and C), multiple cysts of the right kidney (Fig. 1D), hyperechogenic kidneys (Fig. 1E), and hyperechoic mass in the right lung (Fig. 1F) were observed. In retrospect, the first-trimester screening results (nuchal translucency, NT, 1.6 mm) was normal. The AFP MOM value of down's screening in the second trimester was 2.46 mom. There is no family history for genetic disorders and the couple was nonconsanguineous. A amniocentesis was performed to perform fetal chromosomes and genome-wide copy number variants after informed consent. The couple was non-consanguineous and had no family history for genetic disorders. The fetal karyotype was 46,XY (Fig. 2). In addition, the result of CNV-seq showed a 0.26-Mb deletion segment at Xq26.2 (132800000–133060000) involving exon 3 to 6 of the GPC3 gene (Fig. 3). The microdeletion was subsequently confirmed from the mother, and the father was normal. Finally, the couple decided to terminate the pregnancy after receiving adequate genetic counseling. Patient has provided informed consent for publication of the case. This study was approved by the institutional ethics committee of Xuzhou Central Hospital (XZXY-LJ-20190210-037).

## 3 Discussion

SGBS1 characterized by overgrowth of multiple systems was first described in 1975 by Simpson.^[4]^ It has obvious clinical heterogeneity, in addition to the typical features of SGBS1, also presenting with various degrees of mental retardation and embryonal tumors.^[5]^ The pathogenesis of SGBS1 is featured by mutations or intragenic deletions/duplications in the GPC3 and GPC4 gene localized at chromosome Xq26.^[6–8]^ Glypicans play a role in the regulation of growth factor activity.^[9]^ The majority of the mutations in the reported cases originate from the mother, and the minority are de novo.^[10]^ In the present case, a fetus was presented with markedly developed overgrowth, congenital cardial vascular anomalies, multiple cysts of the right kidney and hyperechoic alteration, and abnormal mass in the right lung. The further amniocentesis was conducted and amniotic fluid cells were cultured, simultaneously parental chromosomes were verified by CNV-seq. A 0.26-Mb microdeletion segment at Xq26.2 inherited from mother was identified through conventional karyotyping combined CNV-seq. The deletion fragment covering exon 3 to 6 of the GPC3 gene has contributed to the genetic cause of the affected fetus who showed overgrowth, cardiacal structure changes and urinary abnormalities, it was thus diagnosed to be SGBS1. And the pregnancy was ultimately terminated according to the parent's option.

However, prenatal diagnosis for the majority of SGBS1 are relatively difficult due to atypical fetal phenotypes. Fetal macrosomia revealed during pregnancy should needs to be differentiated from other associated disease such as Sotos syndrome, Beckwith-Wiedemann syndrome, Sotos syndrome, and Pallister-Killian syndrome.^[11,12]^ In addition, it has been proposed that elevated maternal plasma alpha-fetoprotein and increased NT may be prenatal markers of SGBS1.^[13,14]^ However, in our case, the fetus displayed normal NT and maternal plasma alpha-fetoprotein. In a review for prenatal symptoms of SGBS1, it was described that fetal macrosomia was the most common abnormality (86%), polyhydramnios accounted for 70% of cases, organomegaly was reported in 60% of cases, followed by renal malformations (32%), congenital diaphragmatic hernia (30%), and cardiac malformations (13%).^[11]^ In our case, the fetus did not express the common prenatal clinical features of polyhydramnios and organomegaly. Therefore a definite diagnosis for prenatal SGBS1 relies on the dynamical fetal detailed information from imageological examination including ultrasound and magnetic resonance, meanwhile, appropriate genetic testing is also necessary to help the clinician determine the diagnosis.

As mentioned above, SGBS1 is an X-linked recessive disorder characterized by mutations in the GPC3 and a few in the GPC4 gene.^[15]^ As is known, the Xq26.2 region is a critical region for regulation of body size, among which GPC3 is involved in the regulation of signaling pathways associated with cell division and growth, such as the signaling of Wnts and Hedgehogs.^[16]^ The loss of functional GPC3 probably leads to hyperactivation of Wnt and Hedgehog signalings, leading to the overgrowth and increased tumor risk.^[17]^ Although all types of GPC3 mutation were found, the most prevalent type was point mutation, followed by large deletions, large duplications and translocations.^[18]^ So when SGBS1 is considered and CNV-seq is negative, whole exon sequencing should be provided. In our case, we confirmed the diagnosis of SGBS1 through genetics results. The 0.26-Mb deletion at Xq26.2 region was detected by CNV-seq. The microdeletion was subsequently verified from the mother. The chance of transmitting the pathogenic variant in each pregnancy is 50%.^[19]^ Males who inherit the pathogenic variant will be affected. Females who inherit the pathogenic variant will be carriers. Carrier females may have mild symptoms, depending on the X-inactivation status.^[6]^ In conclusion, once fetal overgrowth and other malformation are revealed in prenatal ultrasound, although without polyhydramnios and organomegaly, SGBS1 should be considered and further genetic testing such as CNV-seq and whole exon sequencing should be conducted to help clinicians provide a definite diagnosis to guide the following genetic counseling and the next pregnancy.

## Acknowledgments

We thank laboratory staff at Prenatal Diagnosis Medical Center of Xuzhou Central Hospital.

## Author contributions

**Conceptualization:** Jing Sha, Jing-Fang Zhai.

**Data curation:** Fangfang Tan, Ying Liu, Jing Sha.

**Formal analysis:** Fangfang Tan, Jing Sha, Jing-Fang Zhai.

**Funding acquisition:** Jing Sha, Jing-Fang Zhai.

**Supervision:** Fangfang Tan, Jing-Fang Zhai, Xuezhen Wang, Ying Liu, Zaochun Xu.

**Validation:** Fangfang Tan, Zaochun Xu.

**Writing – original draft:** Jing Sha.

**Writing – review & editing:** Jing-Fang Zhai.
